# Supplementary material for: An open-source framework for large-scale, flexible evaluation of biomedical text mining systems
Source: J Biomed Discov Collab. 2008 Jan 29;3:1. doi: 10.1186/1747-5333-3-1 (PMC2276192; doi:10.1186/1747-5333-3-1)
Supplement: Additional file 1 — Evaluation platform source code. The additional file contains the software evaluation framework discussed in this paper. The most current release can be downloaded from SourceForge.net [1]. See the accompanying README file for instructions on installation and use. Also included in the distribution is a collection of UIMA wrappers for some commonly used BioNLP tools and annotated corpora. [file 1747-5333-3-1-S1.zip › bionlp_uima_repository-1.0.1/docs/api/edu/uchsc/ccp/util/nlp/tokenizer/Tokenizer.html]

Tokenizer (Center for Computational Pharmacology Util/UIMA API)


|  |  |  |
| --- | --- | --- |
| **Overview** \| **Package** \| **Class** \| **Tree** \| **Deprecated** \| **Index** \| **Help** | |  |
| PREV CLASS   NEXT CLASS | **FRAMES**    **NO FRAMES**     **All Classes** |  |
| SUMMARY: NESTED \| FIELD \| CONSTR \| METHOD | DETAIL: FIELD \| CONSTR \| METHOD |  |


---


## edu.uchsc.ccp.util.nlp.tokenizer Interface Tokenizer

**All Known Implementing Classes:**: PennBioTokenizer\_Util

---

``` public interface Tokenizer ```

This class is an interface for tokenizers

**Author:**
:   William A Baumgartner, Jr.

---

| **Method Summary** | |
| --- | --- |
| `List<TextAnnotation>` | `getTokensFromString(String inputText, String inputDocumentID)`             Given a `String` of input text, and a documentID, return tokens in the form of `TextAnnotation` objects. |

**Method Detail**

### getTokensFromString

```
List<TextAnnotation> getTokensFromString(String inputText,
                                         String inputDocumentID)
```

:   Given a `String` of input text, and a documentID, return tokens in the form of `TextAnnotation` objects.

    :   **Parameters:**: `inputText` - the text to tokenize: `inputDocumentID` - an identifier for the text being tokenized. This document ID will be associated with each of the `TextAnnotation` objects that are returned. **Returns:**: a List of `TextAnnotation` objects, one for each token


---


|  |  |  |
| --- | --- | --- |
| **Overview** \| **Package** \| **Class** \| **Tree** \| **Deprecated** \| **Index** \| **Help** | |  |
| PREV CLASS   NEXT CLASS | **FRAMES**    **NO FRAMES**     **All Classes** |  |
| SUMMARY: NESTED \| FIELD \| CONSTR \| METHOD | DETAIL: FIELD \| CONSTR \| METHOD |  |


---
